# Comparison of various methods for validity evaluation of QSAR models

**DOI:** 10.1186/s13065-022-00856-4

**Published:** 2022-08-23

**Authors:** Shadi Shayanfar, Ali Shayanfar

**Affiliations:** 1 Student Research Committee, Faculty of Pharmacy, Tabriz University of Medical Sciences, Tabriz, Iran; 2 Pharmaceutical Analysis Research Center, Tabriz University of Medical Sciences, Tabriz, Iran; 3 Editorial Office of Pharmaceutical Sciences Journal, Faculty of Pharmacy, Tabriz University of Medical Sciences, Tabriz, Iran

**Keywords:** Biological activity, External validation, QSAR, Statistical parameters

## Abstract

**Background:**

Quantitative structure–activity relationship (QSAR) modeling is one of the most important computational tools employed in drug discovery and development. The external validation of QSAR models is the main point to check the reliability of developed models for the prediction activity of not yet synthesized compounds. It was performed by different criteria in the literature.

**Methods:**

In this study, 44 reported QSAR models for biologically active compounds reported in scientific papers were collected. Various statistical parameters of external validation of a QSAR model were calculated, and the results were discussed.

**Results:**

The findings revealed that employing the coefficient of determination (r^2^) alone could not indicate the validity of a QSAR model. The established criteria for external validation have some advantages and disadvantages which should be considered in QSAR studies.

**Conclusion:**

This study showed that these methods alone are not only enough to indicate the validity/invalidity of a QSAR model.

**Supplementary Information:**

The online version contains supplementary material available at 10.1186/s13065-022-00856-4.

## Introduction

Quantitative structure–activity relationship (QSAR) is a numerical method for finding the relationships between chemical structure and drug properties i.e., biological activity in drug discovery processes [1]. Developing a QSAR model composed of different stages i.e., (1) collecting data from the literature, (2) calculation of parameters performed by different software packages such as Dragon software or image analysis (2D-QSAR), force field calculations based on three-dimensional structures (3D-QSAR) and etc., (3) developing the QSAR model by various statistical technique e.g. multiple linear regression, artificial neural network and partial least square, and (4) validation of the model by internal (leave one out and leave many out) and external validation [2]. There are various critical points in QSAR studies that should be considered by researchers [3]. However, the challenges on selecting appropriate parameters for external validation have been seen in the literature [4, 5].

In QSAR studies, training a model by linear and non-linear models is not enough to confirm the prediction capability. The developed model should be applied to other data sets which did not synthesize in virtual screening and designing new drug compounds. On the way, whenever we can say a QSAR model is acceptable that it could predict the activity of other compounds with reasonable accuracy. Therefore, external validation (splitting data into training and test sets) is one of the major challenges in QSAR studies [6–8]. Various types of cross validation analysis i.e., leave one out, leave many out and repeated double cross validation are recommended in QSAR studies especially when the available sample size is small [9, 10]. However, external validation is one of the most common criteria for evaluating the validity of a QSAR model [11–13].

Different criteria and rules were proposed for evaluating the validity of the QSAR models, which most of them focused on the external validation [13, 14]. Five criteria proposed in authentic journals were selected in this study and details have been described in method section. They are highly cited and several researchers were used them to evaluate validity of QSAR models [15–18]. Designers of each criterion have been shown advantages of them in comparison with others for external validation of QSAR models [5, 6, 19–21]. Some models have certain defects from the statistical viewpoint and various results are observed based on the applied software e.g. the correlation coefficient (r^2^) of regression through origin [5]. Nevertheless, there is no comprehensive comparison between them for the evaluation of the external validity of QSAR models. The aim of this study is the comparison of external validation of QSAR models by them to find advantages and disadvantages of each method.

## Methods

Forty-four data sets (training and test sets) composed of experimental biological activity and corresponding calculated activity (re-substitution value for training data set) using QSAR models with various statistical approaches were collected from the published articles [22–48] indexed in Scopus database (see Additional file 1 and Table 1). The absolute error (AE) of each datum (absolute difference between experimental and calculated data) was calculated. External validation of these data set was assessed with the following methods:

**Table 1:** The numerical values of statistical parameters which need to calculate mentioned criteria for external validation for 44 developed QSAR models

No.	Number of compounds in training set	Number of compounds in test set	r^2^ > 0.6	$r_{0}^{2}$(Eq. 3)	${\text{r}}_{{0}}^{{^{\prime}2}}$(Eq. 4)	${\text{r}}_{{0}}^{{2}} = {\text{r}}_{{0}}^{{^{\prime}2}}$(Eq. 5)	AEE ± SD Training set	AEE ± SD Test set	Training set range	Refs.
1	39	10	0.917	0.909	0.917	0.999	0.161 ± 0.114	0.221 ± 0.110	4.07	[23]
2	39	10	0.880	0.879	0.857	0.999	0.237 ± 0.234	0.318 ± 0.150	4.07	[23]
3	31	10	0.715	0.715	0.617	0.997	0.167 ± 0.171	0.266 ± 0.244	1.72	[24]
4	26	11	0.725	0.310	0.691	0.997	0.233 ± 0.167	0.354 ± 0.301	2.74	[25]
5	40	10	0.906	0.904	0.904	0.999	0.121 ± 0.091	0.206 ± 0.095	2.68	[26]
6	40	10	0.892	0.879	0.892	0.999	0.122 ± 0.087	0.195 ± 0.146	2.68	[26]
7	68	17	0.261	0.012	0.052	0.957	0.503 ± 0.435	1.165 ± 0.715	5.00	[27]
8	68	17	0.444	0.220	0.404	0.977	0.331 ± 0.674	0.435 ± 0.326	4.60	[27]
9	42	11	0.834	0.823	0.818	0.824	0.872 ± 0.678	1.630 ± 1.256	14.46	[28]
10	42	9	0.588	0.552	0.511	0.999	0.040 ± 0.035	0.169 ± 0.124	1.85	[29]
11	42	9	0.748	0.496	0.730	0.999	0.053 ± 0.043	0.133 ± 0.077	1.85	[29]
12	20	6	0.963	0.962	0.983	0.787	0.052 ± 0.043	0.035 ± 0.035	0.91	[30]
13	90	22	0.372	0.376	-0.292	0.950	0.432 ± 0.648	0.538 ± 0.647	6.95	[31]
14	68	17	0.382	0.136	0.309	0.975	0.364 ± 0.324	0.457 ± 0.356	4.90	[31]
15	27	5	0.088	− 2.263	− 1.129	0.995	0.074 ± 0.094	0.315 ± 0.135	0.40	[32]
16	27	7	0.818	− 1.721	0.563	0.993	0.412 ± 0.352	0.645 ± 0.489	3.76	[33]
17	27	7	0.763	− 4.030	0.462	0.992	0.359 ± 0.290	0.729 ± 0.511	3.76	[33]
18	89	19	0.932	0.932	0.928	0.998	0.187 ± 0.151	0.246 ± 0.204	3.95	[34]
19	89	19	0.821	0.813	0.811	0.995	0.255 ± 0.186	0.339 ± 0.368	3.95	[34]
20	66	16	0.703	0.514	0.914	0.984	0.444 ± 0.338	0.678 ± 0.411	5.45	[35]
21	66	16	0.671	0.475	0.700	0.983	0.384 ± 0.324	0.706 ± 0.461	5.45	[35]
22	66	16	0.914	0.908	0.670	0.995	0.288 ± 0.232	0.297 ± 0.307	5.45	[35]
23	32	11	0.790	0.006	0.683	0.993	0.120 ± 0.094	0.501 ± 0.249	4.68	[47]
24	40	12	0.876	0.875	0.845	0.999	0.090 ± 0.079	0.238 ± 0.088	3.35	[36]
25	40	12	0.866	0.814	0.861	0.999	0.079 ± 0.084	0.205 ± 0.140	3.35	[36]
26	63	16	0.999	0.999	0.999	1.000	0.011 ± 0.006	0.011 ± 0.006	3.76	[37]
27	40	4	0.960	0.693	0.863	1.000	0.155 ± 0.118	0.178 ± 0.105	4.25	[38]
28	22	7	0.995	0.995	0.995	1.000	0.043 ± 0.064	0.046 ± 0.032	2.56	[39]
29	22	7	0.971	0.971	0.971	0.999	0.101 ± 0.127	0.097 ± 0.097	2.56	[39]
30	50	18	0.914	0.796	0.879	1.000	0.041 ± 0.038	0.068 ± 0.134	2.35	[40]
31	50	18	0.994	0.993	0.992	1.000	0.031 ± 0.028	0.026 ± 0.028	2.35	[40]
32	52	12	0.815	0.686	0.801	0.983	0.340 ± 0.269	0.297 ± 0.261	3.32	[41]
33	58	6	0.964	0.949	0.958	1.000	0.051 ± 0.048	0.127 ± 0.117	2.90	[42]
34	58	6	0.966	0.965	0.962	1.000	0.066 ± 0.052	0.105 ± 0.076	2.90	[42]
35	47	16	0.899	0.878	0.898	0.999	0.195 ± 0.117	0.186 ± 0.153	2.16	[43]
36	52	20	0.533	0.367	0.511	0.995	0.566 ± 0.378	0.383 ± 0.314	4.28	[44]
37	52	20	0.659	0.533	0.657	0.997	0.554 ± 0.521	0.327 ± 0.230	4.28	[44]
38	52	20	0.744	0.714	0.733	0.998	0.355 ± 0.343	0.282 ± 0.213	4.28	[44]
39	52	20	0.815	0.785	0.814	0.998	0.290 ± 0.358	0.246 ± 0.181	4.28	[44]
40	31	10	0.658	0.475	0.658	0.995	0.097 ± 0.064	0.272 ± 0.202	2.17	[45]
41	68	8	0.898	0.865	0.935	0.999	0.125 ± 0.110	0.204 ± 0.151	4.03	[46]
42	68	8	0.855	0.702	0.828	0.998	0.199 ± 0.115	0.270 ± 0.148	4.03	[46]
43	53	18	0.806	0.678	0.795	0.996	0.122 ± 0.118	0.279 ± 0.203	3.78	[48]
44	53	18	0.676	0.109	0.640	0.993	0.329 ± 0.271	0.362 ± 0.276	3.78	[48]

### Proposed criteria by Golbraikh and Tropsha

I. r^2^ > 0:6, r^2^ is the coefficient of determination between the experimental activity and predicted values based on regression analysis.

II. 0.85 < K < 1.15 or 0.85 < K' < 1.15.

K and K' are slopes of regression lines through the origin between the experimental activity and predicted, and vice versa, respectively.

III. $$\frac{{\text{r}}^{2}-{\text{r}}_{0}^{2}}{{\text{r}}^{2}}\text{<0.1 or }\frac{{\text{r}}^{2}-{\text{r}}_{0}^{^{\prime}2}}{{\text{r}}^{2}}\text{<0.1}$$

r_0_.^2^ and ${\text{r}}_{0}^{^{\prime}2}$ is the coefficient of determination between the experimental activity and predicted values and predicted versus experimental activity, respectively, based on regression through origin analysis (linear regression by least square method without a constant term) [19].

### Proposed criteria by Roy based on regression through origin (RTO)

Roy and coworkers suggested ${\text{r}}_{{\text{m}}}^{{2}}$ which calculated by Eq. 1, and it is one of the most famous equations which used by QSAR experts in literature [20, 49]:

1 $$r_{m}^{2} = r^{2} \left( {1 - \sqrt {r^{2} - r_{0}^{2} } } \right)$$

In this equation,$r_{0}^{2}$ value computed using regression through origin (RTO) and RTO referred to linear regression by least square method without a constant term.

### Concordance correlation coefficient (CCC)

Gramatica and coworker [4] suggested the concordance correlation coefficient (CCC) for external validation of a QSAR model:

2 $${\text{CCC}} = \frac{{2\sum\limits_{{{\text{i}} = 1}}^{{{\text{n}}_{{{\text{EXT}}}} }} {\left( {{\text{Y}}_{i} - \overline{{\text{Y}}} } \right)\left( {{\text{Y}}_{{{\text{i}}^{\prime } }} - \overline{{\text{Y}}}_{{{\text{i}}^{\prime } }} } \right)} }}{{\sum\limits_{{{\text{i}} = 1}}^{{{\text{n}}_{{{\text{EXT}}}} }} {\left( {{\text{Y}}_{{\text{i}}} - \overline{{\text{Y}}} } \right)^{2} } + \sum\limits_{{{\text{i}} = 1}}^{{{\text{n}}_{{{\text{EXT}}}} }} {\left( {{\text{Y}}_{{{\text{i}}^{\prime } }} - \overline{{\text{Y}}}_{{{\text{i}}^{\prime } }} } \right)^{2} + {\text{n}}_{{{\text{EXT}}}} \left( {{\text{Y}}_{{{\text{i}}^{\prime } }} - \overline{{\text{Y}}}_{{{\text{i}}^{\prime } }} } \right)^{2} } }}$$

Y_i_ is the experimental value, $\mathop {\text{Y}}\limits^{ - }$ is the average of experimental values, ${\text{Y}}_{{{\text{i}}^{\prime } }}$ is the predicted value of activity and ${\overline{\text{Y}}}_{{\text{i}}}$ is the average of the predicted value of the activity. EXT is external prediction set or test set. CCC > 0.8 accounts as a valid model.

### Statistical significant between deviation of experimental activity and calculated data

In 2014, our research group challenged the regression through origin and proposed the calculation of model errors for training and test sets and comparison of them as a reliable method to external validation of QSAR models [5].

### Criteria based on training set range and the deviation between experimental and calculated data

Roy and coworkers [21] similar to our method (method 4) proposed new principles based on training set range and absolute average error (AAE) i.e., the difference between experimental and the predicted values of test set, and corresponding standard deviation (SD) for training and test sets as follows:

Good prediction: AAE ≤ 0.1 × training set range and AAE + 3 × SD ≤ 0.2 × traning set range

Bad prediction: AAE > 0.15 × training set range or AAE + 3 × SD > 0.25 × traning set range

A good model should be passed both above criteria. However, the predictions which fall into one of the conditions could be considered as of moderately acceptable model.

## Results and discussion

Table 1 listed the numerical values of statistical parameters that need to calculate the mentioned criteria for external validation of 44 developed QSAR models.

The main factor in the validation of QSAR models from a statistical point is different equations even to calculate simple parameters such as r^2^ and r_0_^2^ [22, 50]. These different equations will affect the comparison. The r^2^ in this work was calculated by SPSS software based correlation between experimental and calculated values. However, in the studied criteria in this work, there is a controversy in the calculation of r_0_^2^. The following equations were applied to the calculation of r_0_^2^ and in method 1, 2 and Excel software [21]

3 $${\text{r}}_{0}^{2} = 1 - \frac{{\sum {\left( {Y_{i} - \left( {Y_{fit} = KY_{{i^{\prime}}} } \right)} \right)^{2} } }}{{\sum {\left( {Y_{i} - \overline{Y}_{i} } \right)^{2} } }}$$

4 $${\text{r}}_{{0}}^{{^{\prime}2}} = 1 - \frac{{\sum {\left( {{\text{Y}}_{{\text{i}}} - \left( {{\text{Y}}_{{{\text{fit}}}} = {\text{K}}^{\prime } \;{\text{Y}}_{{{\text{i}}^{\prime } }} } \right)} \right)^{2} } }}{{\sum {\left( {{\text{Y}}_{{\text{i}}} - \overline{{\text{Y}}}_{{\text{i}}} } \right)^{2} } }}$$

Instead, the alternative formula was proposed instead of the Eqs. 3 and 4 because of statistical defects to the calculation of r^2^ of RTO [5, 22] which recommended by statistical books in the literature [51, 52]:

5 $${\text{r}}_{{0}}^{{2}} = {\text{r}}_{{0}}^{{^{\prime}2}} { = }\frac{{\sum {\mathop {{\text{Y}}_{{{\text{fit}}}}^{{2}} }\limits^{{}} } }}{{\sum {\mathop {{\text{Y}}_{{\text{i}}}^{{2}} }\limits^{{}} } }}$$

In addition to statistical defects in Eq. (3) and (4) for the calculation of r_0_^2^ and r_0_^′2^, QSAR researchers, may apply Eq. (5) which proposed as an appropriate equation for r_0_^2^ and officinal statistical package such as SPSS, and do not give reasonable results. Calculation of ${\text{r}}_{{\text{m}}}^{{2}}$ based on computed $r_{0}^{2}$ by Eq. (5) (or SPSS software) is not possible because of r^2^ is commonly less than $r_{0}^{2}$ and therefore ${\text{r}}^{{2}} {\text{ - r}}_{{0}}^{{2}} { < 0}$. This is the most defect of methods 1 and 2 for the external validation of QSAR models.

Seven of the studied models have r^2^ < 0.6 (Table 2). Therefore, they could not account as valid models. r^2^ is simple parameter to evaluate the correlation between experimental and predicted values in QSAR studies and for estimating the correlation between concentration and response in analytical chemistry. It is a primary criterion, and a QSAR model or a developed analytical method with a high r^2^ value does not necessarily have an acceptable validity [53, 54]. In addition, the squared factors e.g. r^2^, negatively affects the possibility to distinguish errors in one or in another direction: overpredicted or underpredicted values; these two kinds of errors have a huge different in toxicity and regulatory evaluation.

**Table 2:** Values of the proposed criteria (method 1–5) for external validation of QSAR models

Model	Method 1					Method 2	Method 3	Method 4	Method 5					
	r^2^ > 0.6	0.85 < K or K´ < 1.15		$\frac{{\text{r}}^{2}-{\text{r}}_{0}^{2}}{{\text{r}}^{2}}\text{ or }\frac{{\text{r}}^{2}-{\text{r}}_{0}^{^{\prime}2}}{{\text{r}}^{2}}\text{<0.1}$		$r_{m}^{2}$ > 0.5	CCC > 0.8	p-value	I^b^	II^c^	III^d^	IV^e^	V^f^	VI^g^
1	0.917	1.00	1.00	0.010	0.000	0.83	0.95	0.14	0.55	0.41	0.81	0.61	1.02	G
2	0.880	1.01	0.98	0.000	0.025	0.86	0.92	0.31	0.77	0.41	0.81	0.61	1.02	G
3	0.715	1.00	1.00	0.000	0.138	0.71	0.84	0.18	1.02	0.17	0.34	0.26	0.43	B
4	0.725	0.98	1.02	0.573	0.047	0.26	0.77	0.23	1.26	0.27	0.55	0.41	0.69	B
5	0.906	1.00	1.00	0.002	0.003	0.86	0.95	0.01	0.49	0.27	0.54	0.40	0.67	G
6	0.892	1.00	1.00	0.015	0.000	0.79	0.94	0.16	0.63	0.27	0.54	0.40	0.67	M
7	0.261	0.98	0.98	0.956	0.800	0.13	0.51	< 0.01	3.31	0.50	1.00	0.75	1.25	B
8	0.444	0.97	1.01	0.506	0.090	0.23	0.66	0.543	1.41	0.46	0.92	0.69	1.15	B
9	0.834	0.74	1.11	0.014	0.020	0.75	0.89	0.08	5.40	1.35	2.70	2.02	3.37	B
10	0.588	1.02	0.98	0.062	0.131	0.48	0.68	0.01	0.54	0.19	0.37	0.28	0.46	B
11	0.748	0.98	1.02	0.336	0.024	0.37	0.75	0.01	0.36	0.19	0.37	0.28	0.46	G
12	0.963	1.05	0.92	0.001	− 0.021	0.93	0.97	0.41	0.14	0.09	0.18	0.14	0.23	G
13	0.372	1.00	0.95	− 0.012	1.786	ND^a^	0.57	0.49	2.48	0.70	1.40	1.05	1.74	B
14	0.382	1.01	0.97	0.644	0.189	0.19	0.61	0.30	1.53	0.49	0.98	0.74	1.23	B
15	0.088	1.02	0.97	26.745	13.844	− 0.05	− 0.25	< 0.01	0.72	0.04	0.08	0.06	0.10	B
16	0.818	1.05	0.95	3.105	0.312	− 0.49	0.55	0.16	2.11	0.38	0.75	0.56	0.94	B
17	0.763	1.05	0.94	6.282	0.394	− 0.91	0.43	0.02	2.26	0.38	0.75	0.56	0.94	B
18	0.932	1.01	0.99	0.000	0.004	0.92	0.96	0.14	0.80	0.40	0.79	0.59	0.99	M
19	0.821	1.01	0.98	0.009	0.012	0.75	0.90	0.34	1.44	0.40	0.79	0.59	0.99	B
20	0.703	0.97	1.01	0.270	− 0.300	0.40	0.81	0.02	1.91	0.55	1.09	0.82	1.36	B
21	0.671	0.96	1.02	0.292	− 0.044	0.37	0.79	0.02	2.09	0.55	1.09	0.82	1.36	B
22	0.914	0.99	1.03	0.007	0.268	0.84	0.95	0.90	1.22	0.55	1.09	0.82	1.36	M
23	0.790	0.91	1.09	0.992	0.136	0.09	0.60	< 0.01	1.25	0.47	0.94	0.70	1.17	B
24	0.876	1.00	1.00	0.002	0.035	0.84	0.93	< 0.01	0.50	0.34	0.67	0.50	0.84	G
25	0.866	0.99	1.01	0.059	0.005	0.67	0.92	0.01	0.62	0.34	0.67	0.50	0.84	G
26	0.999	1.00	1.00	0.000	0.000	1.00	1.00	0.65	0.03	0.38	0.75	0.58	0.94	G
27	0.960	0.98	1.02	0.278	0.101	0.46	0.83	0.72	0.03	0.43	0.85	0.64	1.06	G
28	0.995	1.00	1.00	0.000	0.000	0.99	1.00	0.90	0.14	0.26	0.51	0.38	0.64	G
29	0.971	1.00	1.00	0.000	0.000	0.96	0.99	0.93	0.39	0.26	0.51	0.38	0.64	G
30	0.914	1.00	1.00	0.129	0.038	0.60	0.93	0.42	0.47	0.24	0.47	0.35	0.59	M
31	0.994	1.00	1.00	0.002	0.002	0.96	1.00	0.51	0.11	0.24	0.47	0.35	0.59	G
32	0.815	1.03	0.95	0.158	0.017	0.52	0.87	0.61	1.09	0.33	0.66	0.50	0.83	B
33	0.964	1.01	0.99	0.016	0.006	0.85	0.96	0.18	0.48	0.29	0.58	0.44	0.73	G
34	0.966	1.00	1.00	0.001	0.004	0.94	0.98	0.10	0.33	0.29	0.58	0.44	0.73	G
35	0.899	1.02	0.98	0.023	0.001	0.77	0.91	0.81	0.64	0.22	0.43	0.28	0.54	B
36	0.533	1.01	0.98	0.311	0.041	0.32	0.71	0.06	1.33	0.43	0.86	0.64	1.07	B
37	0.659	1.00	1.00	0.191	0.003	0.43	0.80	0.07	1.02	0.43	0.86	0.64	1.07	M
38	0.744	1.00	1.00	0.040	0.014	0.62	0.86	0.38	0.92	0.43	0.86	0.64	1.07	M
39	0.815	1.01	0.99	0.037	0.001	0.67	0.89	0.50	0.79	0.43	0.86	0.64	1.07	G
40	0.658	0.97	1.03	0.278	0.000	0.38	0.77	0.02	0.88	0.22	0.43	0.33	0.54	B
41	0.898	0.99	1.01	0.037	− 0.041	0.73	0.94	0.03	0.66	0.40	0.81	0.60	1.01	G
42	0.855	1.00	1.00	0.179	0.032	0.52	0.89	0.06	0.71	0.40	0.81	0.60	1.01	G
43	0.806	1.00	0.99	0.159	0.014	0.52	0.87	0.01	0.89	0.38	0.76	0.55	0.95	M
44	0.676	0.99	1.00	0.838	0.053	0.17	0.74	0.66	1.19	0.38	0.76	0.55	0.95	B

^a^$${\text{r}}^{2}\text{<}{\text{r}}_{0}^{2}$$

^b^AAE + 3 × SD

^c^0.1 × training set range

^d^0.2 × training set range

^e^0.15 × training set range

^f^0.25 × training set range

*G* good, *MG* moderately good, *B* Bad

The numerical values of other proposed criteria in method 1 show that all models have K or K' between 0.85 and 1.15. The third rule ($\frac{{\text{r}}^{2}-{\text{r}}_{0}^{2}}{{\text{r}}^{2}}\text{<0.1 or }\frac{{\text{r}}^{2}-{\text{r}}_{0}^{^{\prime}2}}{{\text{r}}^{2}}\text{<0.1}$) is only non-acceptable for 7 models which 3 of them have r^2^ < 0.6. Therefore, based on the suggested principles in method 1, 11 models are not valid.

Method 2 proposed based on RTO and r_0_^2^ calculated by Eq. (3). Twenty-six models have ${\text{r}}_{{\text{m}}}^{{2}}$ > 0.5, and the results are similar to method 1 (both of them are based on RTO). The valid models based on method 1 with r^2^ > 0.75 have ${\text{r}}_{{\text{m}}}^{{2}}$ > 0.5 except model 27 with r_0_^2^ = 0.101 (close to threshold, 0.1).

The third studied method was proposed by Gramatica and named CCC [4]. Twenty-nine models have CCC > 0.8. All of them are valid models based on method 1. The results of methods 2 and 3 are very similar. Two models (20 and 27) only have CCC > 0.8 while the defined values near to threshold i.e., 0.4 < ${\text{r}}_{{\text{m}}}^{{2}}$ < 0.5. Method 3 is comparable to developed methods based on RTO. However, it has not statistical defects and non-identical datum for r_0_^2^ based on proposed equations (Eq. (3) and (4) or Eq. (5)) or software (e.g. Excel or SPSS).

**Fig. 1:**
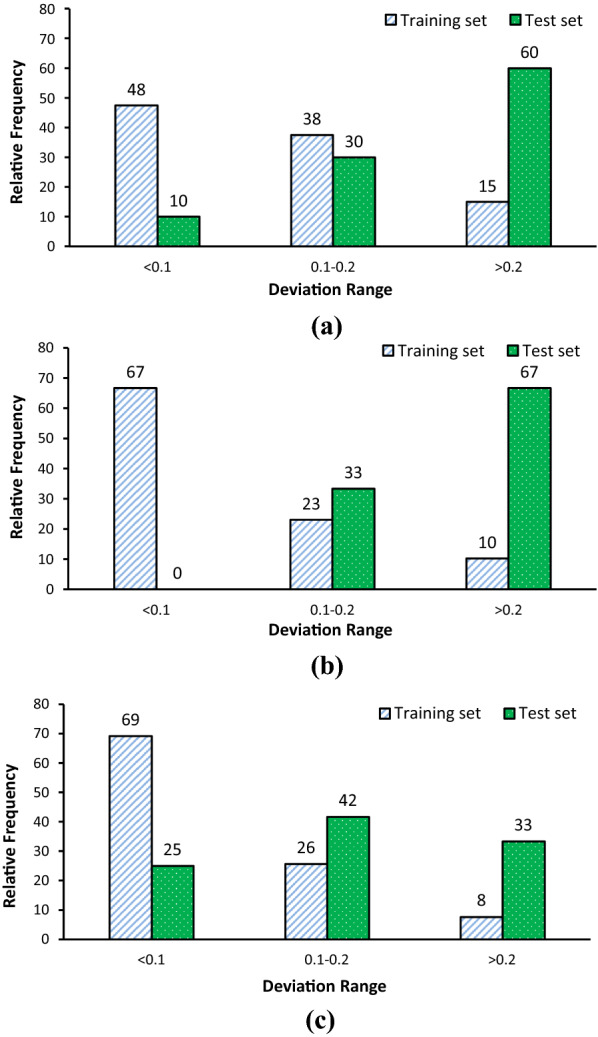
Relative frequency of individual deviation (absolute error) for model 5 (**a**), model 24 (**b**) and model 25 (**c**)

**Fig. 2:**
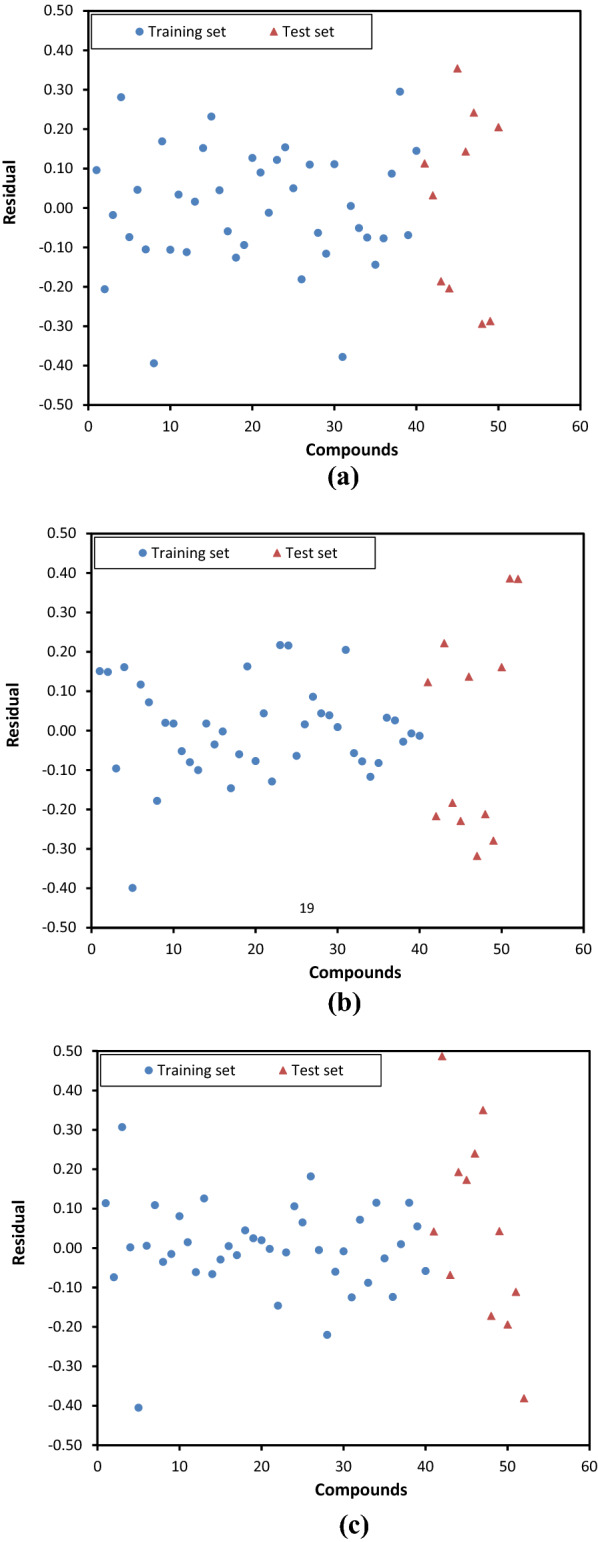
Residual plots for model 5 (**a**), model 24 (**b**) and model 25 (**c**)

Method 4 is based on the calculation of model errors for training and test sets and compares them as a possible reliable method to external validation for models with r^2^ > 0.6 for test set. The aim of developing a QSAR model is the prediction and elucidation of mechanisms of drug action. It is obvious that the prediction capability of training and test sets should be identical. Without considering the training set, it possible statistical parameters for external validation of test set could be acceptable but a significant difference (independent t-test) between prediction power of training and test set might be a weakness for the model. Twenty-six models have r^2^ > 0.6 and no significant difference between absolute error (AE) of training and test sets (p > 0.05). Twenty-three models of them have been selected by CCC as a valid model (CCC > 0.8 and p > 0.05). Model 16 has a CCC = 0.55, and AAE of training and test sets are 0.412 ± 0.352 and 0.645 ± 0.489 (p = 0.16), respectively. High values for SD because of outlier data, is the possible reason for non-significant difference between AEs and it could not account validity of the developed model. On the other hand, models 5, 24 and 25 have CCC > 0.9 and p < 0.01. The relative frequencies of AEs for models 5, 24 and 25 sorted in three subgroups, < 0.1, 0.1–0.2 and > 0.2 and illustrated in Figure 1. In these models, AAE values are low; however, there is 50–250% difference between AAE of training and test sets. On the other hand, in model 5, 48% of the training set and 10% of test sets have AE less than 0.1 while 15% of the training set and 60% of test set have AE more than 0.2. Similar patterns are observed in models 24 and 25. In addition, for those models, residual plots have been illustrated in Figure 2. These plots confirm that there is a significant difference between the prediction capability of developed models for training and test sets and it could not be acceptable for a QSAR model to approve prediction capability.

The last method (method 5) proposed by Roy’s research group based on the training set range and mean and standard deviation of test set data [21]. The models could be classified as GOOD, MODERATELY GOOD and BAD according to their proposed parameters. Most of the models were categorized as BAD (45%) and GOOD (39%) and a few models were MODERATELY GOOD models (Table 2). The first point that should be considered is r^2^ > 0.6 as a necessary criterion. All models which have r^2^ < 0.6 classified as BAD model. Moreover, a good correlation is observed between CCC and GOOD model based on method 5. However, model 11 is a GOOD model while CCC = 0.75 and there is a significant difference between AE of training and test set (AAE of training and test sets are 0.05 and 0.13, respectively and p = 0.01). In comparison with method 4, models 5, 24 and 25 (GOOD models) have a vast difference between AAE of training and test set (Figure 1), although the proposed principles in method 5 could not detect it. A model with a statistically significant difference between the AE of training and test sets might not confirm developing a valid model.

Furthermore, model 3 is a BAD model while CCC = 0.84 and p-value for the difference between AE of training and test is 0.18. AAE of the training set is 0.167 ± 0.171 and 0.266 ± 0.244 (AE ± SD), respectively. High values for SD of training and test sets indicate that there are outlier data which could be considered using statistical parameters e.g. SD of mean errors, in the external validation of QSAR models.

Typographic errors and un-uniformity of applied data set for QSAR modeling or mistake in the determination of the biological activity of studied compounds are a common reason for outlier data, which can decrease the prediction capability of a model. Docking study of outlier cases and comparison with other compounds can help researchers to detect outlier data in developing a QSAR model [55].

These results confirm the results of previous studies which more than a single criterion is recommended to assess the real external predictivity of QSAR models [56]. Moreover, other recommended guidelines in developing QSAR models such as cross validation, appropriate splitting training and test sets variable allocation and correlation coefficients adjusted by degrees of freedom, are other important issues in QSAR studies which should be considered by researchers [10, 57–59]. In addition, cross (internal) validation analysis e.g., leave many out and leave one out are recommended in QSAR studies especially when the sample size is small [9, 10], and some reports showed its superiority in external validation [60]. Therefore, both internal and external validation analysis with considering various criteria are necessary to check the validity of a QSAR model.

## Conclusion

The aim of developing a QSAR model is an acceptable prediction of activity of a compound before synthesis and biological evaluation. Therefore, external validation is necessary. All of the developed methods for external validation of a QSAR model are useful and a good correlation was observed between the studied methods for the selected models. However, some differences were detected between established methods. Methods 1 and 2 are valuable but they are some questionable points in the applied equation for $r_{0}^{2}$ calculation. CCC is a valuable parameter, though in some cases, it cannot detect outlier data. Similar to methods 1 and 2, training data set are not included in CCC. Method 4 and 5 established based on training and test sets. They detected most invalid models, but method 5 considered some model as a GOOD model while the difference between AE of training and test sets are substantial (p < 0.05). On the other way, high SD value in both of training and test sets may pass proposed criterion of method 4 while accounted as a invalid model because of outlier data in training and test sets. Finally, evaluation of a model with either established method is useful, but they did not necessarily mean validity/invalidity of a QSAR model. The results of this study show the importance of calculation error of training and test sets and detection of outliers for checking the validity of a model.

## Supplementary Information

**Additional file 1.** Forty-four data sets (training and test sets) composed of experimental biological activity and corresponding calculated activity.

## Data Availability

All data is available as supplementary.

## Abbreviations

QSAR: Quantitative structure–activity relationship
AE: Absolute error
RTO: Regression through origin
CCC: Concordance correlation coefficient
AAE: Absolute average error
SD: Standard deviation
